# From *Freedom From* to *Freedom To*: New Perspectives on Intentional Action

**DOI:** 10.3389/fpsyg.2019.01193

**Published:** 2019-05-28

**Authors:** Sofia Bonicalzi, Patrick Haggard

**Affiliations:** ^1^ Fakultät für Philosophie, Wissenschaftstheorie und Religionswissenschaft, Ludwig-Maximilians-Universität, Munich, Germany; ^2^ Institute of Cognitive Neuroscience, University College London, London, United Kingdom; ^3^ Laboratoire de Neurosciences Cognitives, Département d’Études Cognitives, École Normale Supérieure, Paris, France; ^4^ Institute of Philosophy, School of Advanced Study, University of London, London, United Kingdom

**Keywords:** intentional action, causal theory, epiphenomenalism, action control, freedom to

## Abstract

There are few concepts as relevant as that of intentional action in shaping our sense of self and the interaction with the environment. At the same time, few concepts are so elusive. Indeed, both conceptual and neuroscientific accounts of intentional agency have proven to be problematic. On the one hand, most conceptual views struggle in defining how agents can adequately exert control over their actions. On the other hand, neuroscience settles for definitions by exclusion whereby key features of human intentional actions, including goal-directness, remain underspecified. This paper reviews the existing literature and sketches how this gap might be filled. In particular, we defend a gradualist notion of intentional behavior, which revolves around the following key features: autonomy, flexibility in the integration of causal vectors, and control.

## Introduction

The selection, planning, and execution of intentional actions are central to our inner sense of self and the interaction with the external world. It thus comes as no surprise that the notions of *intentional action* and *agency* have attracted a considerable share of attention both in philosophy and in cognitive science. During the last few years, a cross-disciplinary naturalistic approach to intentional action – whereby the reference to foundational issues and up-to-date scientific findings are both held to be crucial – has taken hold and progressively flourished (Jeannerod, 2006; Nanay, 2013; Grünbaum, 2017; Mylopoulos and Pacherie, 2018; Haggard, 2019).

With this article, we aim to contribute to an integrated comprehension of agency-related phenomena, paving the way for a shared definition of intentional action. Methodologically speaking, we argue in favor of a view that bridges the gap between disciplinary approaches and promotes a multifaceted understanding of agency.

Both in philosophy and in neuroscience, interest in the topic stems from two intertwined, but plausibly distinguishable, perspectives, which we define as the *subjective* and the *objective* side of agency. The former can be referred to as concerning the subjective feeling surrounding self-attributions of agency. This subjective or phenomenological side of agency has been extensively reviewed elsewhere (Moore and Obhi, 2012; Grünbaum, 2015; Braun et al., 2018). Therefore, we will only briefly touch upon it (see the section “The Subjective Side of Agency”). The latter, which is the target of the present article, refers to the enabling conditions for intentional agency, i.e., the behavioral and neurophysiological features that qualify an action as an intentional one, besides the agent’s distinctive experience accompanying action initiation and outcome production.

Isaiah Berlin famously distinguished between two basic concepts of freedom, namely *freedom from* or negative freedom, and *freedom to* or positive freedom. *Freedom from* consists in the absence of obstacles or constraints to one’s own action. By contrast, *freedom to* identifies the possibility to autonomously determine and achieve individual or collective purposes (Berlin, 1969). In the context of the present article, we borrow Berlin’s distinction in view of discussing two different approaches to intentional action. Recent cognitive neuroscience of action often considers intentional actions as movements that are not triggered by external stimuli, and are therefore internally generated (Passingham et al., 2010). The intentional action is thus *free from* the external constraints represented by the stimulus and the movements to which it leads. In that sense, cognitive neuroscience has to date aligned mostly with *freedom from*. A positive account of intentional action, based on *freedom to*, remains underspecified in the current cognitive neuroscience literature. This article sketches how this gap might be filled (see the section “The Objective Side of Agency”).

In the philosophy of action, the concepts of *intentional action* and *action* are often treated as synonyms. In this review, we examine intentional action in the wider context of voluntary behavior. In particular, we suggest that awareness of the intentions that are seen as causes of one’s own behavior may represent the distinctive feature of intentional action with respect to other forms of voluntary behavior. In the section “The Causal Theory of Action,” we introduce the causal theory of action comparing it to non-compositional accounts of action (see the section “An Anti-compositionalist Understanding of Action”). Building on this, in the section “A Gradualist Understanding of Action” and “The Temporal Structure of Intentions,” we defend a gradualist but still causal understanding of action. In the section “Are Conscious Mental States Causally Relevant?,” we address the question about whether full-fledged intentional action requires intentions to have a causal role at the time of action initiation, by discussing Libet’s experiments on the timing of conscious will. While addressing some criticism to Libet-type experiments, we suggest that an active role for intentions is not necessarily tied to action initiation. By overcoming the simplistic difference between internally and externally generated actions (see the section “Internally Generated and Externally Triggered Actions”), we argue for a multilayered understanding of intentional actions whereby intentional actions are characterized by autonomy (see the section “Intentional Actions and Autonomy”), flexibility in the integration of causal vectors (see the section “Intentional Actions and Causality”), and control (see the section “Intentional Actions and Control”).

## The Subjective Side of Agency

The most striking feature of intentional actions might be of a phenomenological nature. Intentional actions are accompanied by a distinctive sense of agency, whose presence is seen as necessary for somebody to qualify as an agent and not just as a physical cause of an outcome (Gallagher, 2007). The sense of agency accompanying intentional actions has been defined as the sense of being in control of our actions and, through them, of their consequences in the outside world (Haggard, 2008). Individuals are generally expected to have an immediate intuitive appreciation of whether they authored an action or not, despite possible ambiguities at the level of individual causal roles (Blakemore et al., 2003). To give an example, if Peter is part of a platoon shooting a prisoner, he might doubt whether his bullet reached the target and whether his gun contained a real bullet or the blank that is conventionally put in one of the rifles used. However, he is likely to know whether he pulled the trigger or not. He will have a sense of agency with respect to firing the gun if he pulls the trigger, and he will not have this sense if he does not pull the trigger.

The sense of agency has been further analyzed in terms of a *feeling of agency* (FoA), which is a preconceptual low-level experience not necessarily reaching the level of consciousness, and a *judgment of agency* (JoA), which is based on high-level cognitive processes, possibly linked to causal attributions (Synofzik et al., 2008).

The subjective sense of agency can be measured *via* explicit and implicit measures. In experimental paradigms adopting explicit measures, participants are required to evaluate their past performances, e.g., reporting their feeling of control over the outcome of a process or judging whether they authored an action or not. The assumption is that subjects will make the requested evaluation depending on their subjective feeling at the time of the action (van den Bos and Jeannerod, 2002). By contrast, implicit measures, including the intentional binding effect and sensory attenuation in self-touch (Pyasik et al., 2019), do not rely on subjective self-assessments of performance, but instead use indirect readouts of the subjective perception of one’s own agency. Implicit measures, or combinations of implicit and explicit measures, are generally preferred to the extent that they are thought to be immune from idiosyncratic and person-level cognitive biases in self-attribution of agency.

The *intentional binding effect* (Haggard et al., 2002) has been indicated as a potential implicit measure for the sense of agency in both healthy individuals and patients with an abnormal perception of agency. Haggard and colleagues showed that, when an action (e.g., key press) is followed by an effect (e.g., auditory tone), participants perceive the temporal interval between the action and the outcome as shorter than it is in reality. Assuming that the subjective perception of causing an outcome has to do with perceived temporal proximity (Hume, 1738/1978; Blakey et al., 2018), the intentional binding effect may hint at the fact that participants implicitly connect the action and the effect in a meaningful causal sequence (Engbert and Wohlschläger, 2007). Crucially, the effect is only produced when participants voluntarily press the key, and absent when the corresponding bodily movement is passively executed. For this reason, the intentional binding effect is considered to be an implicit marker of agency, i.e., the subjective temporal compression is present when intentional processes are at play, and is widely used in experiments investigating volitional (i.e., intentional) processes and causation (Moore and Obhi, 2012; Fereday and Buehner, 2017).

To the extent that the intentional binding effect is an implicit marker of agency, it is not automatically informative about the subjective explicit feeling of agency. First, it is possible, in principle at least, that a subject exhibiting a higher intentional binding effect explicitly reports a lower sense of control, in a given task, than a subject exhibiting a low intentional binding effect. Second, action and outcome might be perceived as close together in time for reasons unconnected with agency, given that a myriad of factors affect human time perception. Indeed, the exact relation between implicit and explicit markers of agency is a matter of dispute (Ebert and Wegner, 2010; Dewey and Knoblich, 2014), and a clear grasp of the mechanisms and computations underlying the sense of agency is still lacking (David et al., 2008). Third, some recent experimental evidence has shown that physical causation, in the absence of intention, may effectively produce a similar binding pattern (Buehner and Humphreys, 2009; Buehner, 2015; Suzuki et al., 2019).

However, some arguments supporting the linkage between the intentional binding effect and the explicit sense of agency can be provided. Experimental factors that have proven to impact on the magnitude of the intentional binding effect are both associated to variations in explicit markers of agency (Chambon et al., 2013) and treated as relevant for conceptual models of intentional action (Nozick, 1969; Kane, 1996; Mele, 2002). These factors include presence of motivations or reasons for acting (Borhani et al., 2017), obedience to an external coercive source of authority (Caspar et al., 2016), availability of multiple options (Barlas et al., 2018), and fluency of the action selection process and outcome monitoring (Sidarus et al., 2017).

In natural settings, an impoverished integration of sense of agency and voluntary behavior is known to characterize several neurological alterations, such as schizophrenia, alien hand syndrome, Parkinson’s disease, and psychogenic disorders (Blakemore et al., 2002; Garbarini et al., 2016; Saito et al., 2017). Patients affected by Tourette’s syndrome routinely engage in movements that are subjectively perceived as “compulsive” or “unwilled” (Rae et al., 2019) but are enabled by the activation of brain areas normally sub-serving the production of voluntary actions, such as the cortico-striato-thalamo-cortical pathways (Bohlhalter et al., 2006).

In laboratory-based settings, evidence from brain stimulation has suggested that, in given circumstances, the sense of agency and the performance of voluntary movements may also come apart. In particular, stimulation of the supplementary and pre-supplementary motor areas in neurosurgical patients can artificially induce a feeling described as the urge to move the corresponding body part (in the absence of any real movement), or produce the performance of bodily movements that patients identify as their own. Instead, controlled stimulation of the primary motor area may lead patients to perceive their own movements as imposed from without (Fried et al., 1991; Desmurget et al., 2009).

By contrast, in healthy subjects, the sense of agency naturally accompanies the ongoing performance of voluntary movements. Such a smooth attunement between actual movements and the sense of agency might depend on the fact that the same neural circuits, including the pre-supplementary motor area (pre-SMA) and the primary motor cortex (M1) (Moore and Obhi, 2012), are seemingly involved in producing both the sense of agency (i.e., the intentional binding effect) and the corresponding bodily movement.

Psychologist Daniel Wegner has provided a series of results aimed at showing that subjects are unable to reliably judge their authorship over actions, mistakenly reporting that they have initiated actions that were in fact produced by others (Wegner, 2002). The overall claim that people are not reliable in their self-attribution of agency is not new. Bandura reported the presence of self-serving biases in the attribution of agency, with people systematically vindicating the authorship of actions with positive results and dismissing that of actions with negative outcomes (Bandura, 1982; Beyer et al., 2017). However, Wegner’s conclusion has a wider significance to the extent that it is not limited to specific instances of intentional behavior. In particular, self-attributions of agency, linking the intention, the action, and the outcome in a meaningful chain of events, would ultimately depend on erroneous inferential processes that take place retrospectively, after outcome presentation (see also Banks and Isham, 2009). The experience of mentally causing an action would be generated after the outcome is produced. The reason why agents embark in the process of attributing causal power to mental events would be due to the need to make sense of their behaviors in relation to the outside world. That *post facto* evaluations have an influence on self-attributions of agency is not surprising. In particular, misattributions of agency may occur in ambiguous circumstances when, for example, more than one agent is participating in the task and potentially contributing to the outcome.

Clearly, independently of their predictive or postdictive origin, self-attributions of agency are extremely valuable to the agent for navigating the environment and modulating future behavior by learning action-outcome contingencies. Furthermore, current evidence is seemingly insufficient to prove that self-attributions of agency are entirely the by-products of inferential processes: not all experimental findings support such a homogenous interpretation. That the sense of agency can be elicited in neurosurgical patients in the absence of bodily movements (see also Matsuhashi and Hallett, 2008) seemingly suggests that the outcome is not strictly needed for the sense of agency and the related experience of intending to act to arise.

## The Objective Side of Agency

In this section of the paper, we focus on the objective side of agency. The aim is to identify the features that make an action an intentional one, besides the subjective feeling of the agent who is involved in action production.

### The Causal Theory of Action

In the philosophy of action, there is a widespread agreement that intentional actions are bodily movements that the agent is able to control and that control is an intrinsically causal notion (Shepherd, 2014). An almost obligatory starting point in terms of conceptual models of action control is the *causal theory of action*, which has been regarded as the classic move for naturalizing intentional agency. According to the causal theory, the action is intentional if it is appropriately caused by conscious mental states, such as desire-beliefs or plans forming the intention to act (Davidson, 1963; Searle, 1983; Dretske, 1988; Bratman, 2000, 2007; Setiya, 2007). Similar bodily movements can therefore count as intentional or not depending on whether they originate from the agent’s intentions. To give an example, if I decide to raise my arm based on my intention to ask the speaker a question, this counts as an intentional action. By contrast, if I move my arm while asleep, this would not count as an intentional action. Intentions are intrinsically goal oriented, in the sense of having an executive role (i.e., bringing about the end-state) and, more controversially (Rooij et al., 2002), a representational content (i.e., a model of the situation, the action and the end-state). The corresponding bodily movement has a functional role in achieving the end-state set out by the relevant intention.

The applicability of the causal theory extends beyond the philosophy of action. The claim that bodily actions are visible means to invisible ends and that we are able to represent alternatives to our actions is crucial from a normative point of view. The premise of our legal systems is that, in most cases at least, people are punished for their actions to the extent that these actions represent the alterable outputs of the conscious intentions they endorse. The intentional actions we are in control of are thus seen as proper targets of responsibility attributions, reward, and punishment (Edwards, 1958; Moore, 1993; Lagnado and Channon, 2008). By contrast, involuntary movements are movements that are not subordinate to the agent’s conscious plan: controlling agency – in the absence of which no one should be held criminally responsible – is “the mind of a man bent on some conscious action” (Hart, 2008, p. 106).

To the extent that the focus is on the intention guiding the action, the specific bodily movement that executes such an intention is of a comparatively little importance. In judging whether a person is morally accountable and liable to (third-party) punishment, it is the culpable action as a whole, and not the specific bodily movement executed by the culprit, which is under scrutiny. This principle finds a nice neural counterpart in Hebb’s theory of *motor equivalence*. According to the theory of motor equivalence, once a goal has been set, there are several different bodily movements that the brain could still select in order to achieve the same result (Hebb, 1949).

The causal view rests on the assumption that the causal relation between mental states and bodily actions can be framed as a relation between events, e.g., James’ having both the wish to hurt Martin and the belief that by slapping Martin in the face he will hurt him cause James to slap Martin in the face. These events might be ultimately reducible to or supervene on physical, i.e., neural, events. The advantage of such an event-causal view consists in the avoidance of a troublesome, potentially dualist reference to an agent (as a substance, in a Cartesian fashion) causing events, which is central to agent-causal accounts of actions (Reid, 1788/2010, but see O’Connor, 2009). In this respect, the event-causal account is seemingly better suited. However, the causal view has also witnessed several shortcomings, possibly leading to its breaking point.

Perhaps the most daunting challenge facing any causalist approach to intentional action is the problem of deviant causal chains (Mele, 1992; Schlosser, 2010; Shepherd, 2014). The problem of deviant causal chains can be framed as follows. According to the causal theory of action, the ability of the agent to control the action is guaranteed by the very same fact that intentional actions are caused by some relevant mental states. However, there are cases in which causality seemingly does not imply control. This is due to some control-undermining event located between the agent’s mental states and the corresponding action taking place.

Here is our version of a deviant causal chain scenario. Imagine that Bill wants to shoot Joe while hiding on a tree. However, during the ambush, the anxious state produced by the wish to kill Joe makes Bill so nervous that he starts trembling and inadvertently falls from the tree, ending up shooting Joe by mistake. Whereas there is a sense in which Bill’s wish to kill Joe caused the very same outcome that Bill intended to bring about, it is clear that Bill lacked control over the action. As a result, the action fails the test for intentionality despite Bill’s conscious mental states having a causal role in action production. The reason for that is that the outcome is not directly caused by the controlling intention, but indirectly caused by the uncontrolled emotional feelings that this intention triggers.

Envisaged by Davidson (Davidson, 1980), deviant causal chains have indeed become the bugbear of any causal account of action. In fact, it remains difficult to single out relevant cases of control-conveying causation, without providing mere *ad hoc* definitions excluding deviant causation by default. The case of deviant causal chains shows that, whereas it is relatively straightforward to outline a *freedom from* version of what intentional actions are not (i.e., causal chains devoid of deviance), a positive definition, spelling out what appropriateness is, is still missing.

### An Anti-Compositionalist Understanding of Action

The problem of deviant causal chains has given many thinkers the license to suggest that the problem of intentional action cannot be solved by resorting to a causal link between mental states and bodily actions. Philosophers who take an anti-compositionalist stance toward intentional action (O’Brien, 2010; Ford, 2011; Levy, 2013, 2015) have made a recent move in this direction. In particular, Yair Levy has suggested that intentional actions must be taken as primitive items in the agent’s psychological ontology. The difference between intentional and non-intentional actions is that intentional actions are bodily movements that we can continue or stop at wish, e.g., Mary can stop or continue raising her arm to ask the speaker a question, but cannot stop or prevent herself from gasping. The suggestion is appealing because it readily taps into a straightforward understanding of the difference between bodily movements we can and we cannot control. Self-control as the capacity to inhibit actions (Mischel et al., 1972) is indeed recognized as a key feature of human cognition (Filevich et al., 2013; Ghosh et al., 2014).

However, we suggest that this model of action control is also not satisfactory. In particular, it fails to explain the source of control characterizing intentional agency with respect to other types of behavior. In particular, Levy’s suggestion is that intentional actions must be treated like desires or beliefs, to the extent that these entities are all primitive items in people’s cognitive ontology. The major concern is that equating intentional actions to mental states, e.g., desires, beliefs, or intentions, may not be helpful in terms of how to solve the problem of control. Beliefs, desires, or even intentions are not meant to be constitutively subjected to conscious control. Whereas James is expected to exercise control over his slapping Martin in the face, he is not subjected to the same requirement for what concerns his inner wish to hurt Martin or his belief that he can hurt Martin by slapping him in the face. This is not a minor concern, to the extent that control, as an intrinsically causal notion, seems to be one of the marks of intentional agency. The capacity to inhibit actions that we are not willing to initiate or continue is clearly an important feature of our capacity to control our behavior, but is not sufficient to provide a *freedom to* characterization of what a controlled action is.

### A Gradualist Understanding of Action

The anti-compositionalist models of intentional action nonetheless pave the way for a plausibly gradualist interpretation of agency, including intentional and voluntary behavior, as a deeply unified human asset. We can argue for this at different levels. At first glance, this depends on the characterization of voluntary action as what agents have the capacity (James can, i.e., is able to, slap Martin in the face) and the opportunity (James can, i.e., has the chance to, slap Martin in the face) to do. The implication seems to be that what counts as a voluntary action for an individual, i.e., someone having the relevant capacity and opportunity (at times), might not count as a voluntary action for a different individual, i.e., someone lacking the relevant capacity and opportunity (at times). This has interesting implications: neurological patients with functional or organic movement disorders may temporarily lack the capacity to control types of bodily movements that healthy controls, or patients themselves at times, have the capacity to control (Hallett et al., 2012).

A second observation is that the same type of event, e.g., walking, may include tokens that count as intentional actions (e.g., consciously walking toward the door) and tokens that count as mere voluntary (e.g., absent-mindedly pacing about the room) or even involuntary movements (e.g., sleepwalking). Mere voluntary behaviors include habits (i.e., repeated automatic behaviors), which have been extensively investigated, especially in animal studies, as clearly distinguishable from goal-oriented or teleological behavior (Dickinson and Weiskrantz, 1985). We suggest that voluntary actions identify guided movements, i.e., distinguishable from involuntary movements (e.g., reflexes or spasms), which may (partially) lack conscious monitoring. This seems indeed the common feature underlying a vast range of behaviors, including minimal actions (Bach, 1978; Proust, 2003) (e.g., scratching yourself), habitual actions that are performed almost unawares (e.g., brushing one’s teeth), or actions we start doing and suddenly forget why (e.g., trying to recall what one came looking for) (Levy, 2013).

The capacity to assess the determinants of one’s own behavior (i.e., to reason about the reasons of one’s own actions before and after the action takes place) and to report one’s own intentions is a distinctive feature of human volitional processes and may help to characterize intentional, with respect to merely voluntary, actions. Such a characteristic of intentional action is often neglected in the cognitive neuroscience of actions and remains difficult to assess. Nonhuman animals are presumably capable to interact with the environment in order to satisfy internal preferences in view of presumptive rewards (Dickinson and Balleine, 2002). The human capacity for voluntary actions seemingly does not represent a radical departure from this general capacity to act based on punishments and rewards. However, intentional actions may represent a subset of voluntary behavior characterized by conscious awareness or monitoring in selecting the goal and in carrying out the corresponding bodily movement.

The almost exclusive focus on intentions as the conscious psychological states triggering actions has often overshadowed the fact that most of our actions do not in fact exhibit the features of full-fledged intentional behavior. To support action control, the relevant intention must occur before the bodily movement, so that it can work as the explanation or the reason for the action, e.g., James slapped Martin in the face because he wanted to hurt him. However, not all actions fit this model. We constantly engage in voluntary actions we can stop at wish had we acquired awareness of them, but where no temporally prior intention is clearly present. Absentminded voluntary bodily movements are clearly difficult to investigate empirically, since experimental paradigms usually require subjects to attend to their actions in order to report what they did.

### The Temporal Structure of Intentions

To defend a causalist approach to action, one has to explain mere voluntary actions as well. One way to do so consists in appealing to the temporal structure of intentions (Bach, 1978; Searle, 1983; Bratman, 1987; Mele, 1992). In particular, Searle distinguishes between *prior intentions*, setting up the action before it actually takes place, and *intentions in action*, continuously present during action performance. Whereas not all actions are preceded by prior intentions, all actions display intentions in action. In this more limited sense, all actions are guided by conscious mental states. In particular, merely voluntary actions are intentional actions lacking prior intentions, but not intentions in action.

The relation between intention and conscious experience is controversial, and slippery. For example, it seems to be the case that mere voluntary actions may lack conscious intentions in action as well. If Mary absent-mindedly paces about the room [Searle’s example of an intentional action not guided by prior intention (Searle, 1983, p. 84)], it is doubtful whether any conscious intention in action is guiding her behavior. Whereas full-fledged intentional behavior is characterized by conscious awareness, other forms of voluntary behavior are guided in the absence of the agent’s conscious awareness. Building on this model, Pacherie (2008, 2015) has operationalized the traditional distinction between prior intentions and intentions in action in terms of different levels of action specification, sub-served by three types of intentions. Distal intentions operate at a highest/more abstract, context-independent, level, by setting up the overarching goal of the action and the appropriate sub-goals that are necessary to reach the overarching goal. Proximal intentions transfer the general action plan, set up at the previous level, into the current situation of action and select the appropriate motor planning. Finally, motor intentions (crucially not necessarily accessible to consciousness), corresponding to motor representations, are in charge of setting the finest parameters needed to execute the action, by using external sensory information.

Motor representations are nonpropositional states representing both the outcome of the action and the kinematic properties of the movement (Proust, 2003; Mylopoulos and Pacherie, 2017, 2018). However, no consensus has been reached about whether motor representations can count as intentions. Moreover, given that motor intentions are nonpropositional states, the problem is to explain how they can effectively interlock with propositional states, e.g., prior intentions, in view of a goal (Butterfill and Sinigaglia, 2014; Brozzo, 2017). A proper causal theory of action should thus offer a complete account of how different levels of intentions can interlock in order to initiate the action and coordinate the bodily movements that are necessary in view of a goal. An understanding of these mechanisms may contribute to cast light on the differences between intentional and mere voluntary actions.

### Are Conscious Mental States Causally Relevant?

Causal views of intentional action share the view that, at least when it comes to purposeful intentional actions, conscious intentions, which might be reducible to or supervene on physical states, must play a causal role in action initiation. In contrast, the cognitive neuroscience of volition and action has made little reference to conscious mental states as the identifying mark of intentionality or voluntariness, or often argued against the causal role of conscious mental states. In the last few decades, the view that conscious intentions play a causal role in action initiation has been fiercely challenged.

In particular, Libet’s results (Libet et al., 1982, 1983; see also Fried et al., 2011) indicate that the timing of the reported conscious awareness of wanting to make a movement is only subsequent to the onset of the *Readiness Potential* (RP), a gradual increase in negativity in the electrical activity in motor areas peaking at the time of the movement (just before muscle contraction). The RP is hardly visible at the level of single trials (but see Schultze-Kraft et al., 2016) but can be extracted from continuous EEG signal by back averaging a number of epochs time-locked to individual self-paced movements. The method was developed by Kornhuber and Deecke (1965), who were the first to report the RP or *Bereitschaftspotential*.

In the classic experiment by Libet, participants were invited to repeatedly execute the same bodily movement (e.g., moving their wrist) at the time of their choice, while mentally noting the timing (with respect to a rotating clock) in which they felt they wanted to make a movement (i.e., they felt the *urge* to make a movement). This time (W) was then communicated to the experimenter. Starting 1 s or more before the onset of the action, the RP shows a wide distribution, with a main localization in the medial frontal cortex. About 500–200 ms before the onset of the movement, the topography shifts [*Lateralised Readiness Potential* (LRP)] toward the hemisphere that is contralateral to the limb that is about to move (Haggard and Eimer, 1999, but see Schlegel et al., 2013 for a failed replication).

The RP has been traditionally considered the signature of planning and preparing the corresponding movement. The absence of the RP when the action is triggered by external signals (Jahanshahi et al., 1995) motivates the association between the RP and the volitional processes leading to action. Whereas the RP indicates the general intention to move, the LRP is associated to the moment when the brain generates the more specific information needed to move one limb (for a different interpretation, according to which W correlates with the peak amplitude of the LRP, i.e., higher peak amplitude of the LRP in early-W subjects than in late-W subjects, see Douglas et al., 2015).

The reason why the presence of the RP challenges the causal efficacy of conscious intentions is that the RP onset is located before W and may potentially cause the intention to act. Clearly, the fact that the RP temporally precedes W does not automatically justify the claim that the RP causes the intention to move and the corresponding movement. However, if the emergence of conscious intentions follows the onset of the RP, the concept of a prior intention setting up the action in view of a goal starts to crumble. *Epiphenomenalism* is the corresponding view that conscious mental states cannot play a causal role in action production (see Nadelhoffer, 2011; Nahmias, 2014). Criticisms to this view, as grounded in Libet-type experiments, tend to focus on (1) the ecological validity and (2) the methodology underlying these findings. Here, we briefly review these criticisms. We suggest that these criticisms appropriately target the limitations of Libet-type experiments without dismissing them as wrongheaded. We suggest that criticizing Libet-type experiments as mistaken might not be the only strategy to preserve intentional action. By contrast, our suggestion is that action initiation might not be the correct *locus* where we should look for the role of conscious intentions in action production.

#### The Ecological Validity of Libet-Type Experiments

The majority of the criticisms touches upon the trade-off between building an empirically tractable model of intentional processes and providing a more valid proxy for the understanding of action. Firstly, it has been argued that Libet-type experiments are inadequate to the scope of investigating actions guided by distal intentions. These actions typically depend on planning, i.e., the execution phase does not immediately follow the deliberation phase (e.g., Nahmias, 2002; Mele, 2010). Planned actions are difficult to explain with reference to mistaken retrospective inferences following action execution. Consider the following case: Peter decides at t_0_ that he will go to Paris at t_1_. According to the causalist model, Peter’s t_0_-desire to go to Paris at t_1_ and his t_0_-belief that, by taking the train from London, he can get to Paris jointly cause him to go to Paris at t_1_, thus constituting a reason for his action. It is unclear how to explain away the presumptive causal power of Peter’s t_0_-desire and t_0_-belief – jointly setting his t_0_-plan to go to Paris and therefore his t_1_-action – by suggesting that only at t_2_, after action execution, he mistakenly infers that his t_0_-desire and t_0_-belief were causally efficacious. Since experiments on volition and actions are often silent with respect to these types of behaviors, they are inadequate to explain planned actions.

However, a way to investigate experimentally the role of distal and immediate intentions was envisioned by Libet’s himself by means of the distinction between Type I and Type II RPs (Libet et al., 1983. See also Verleger et al., 2016). In performing the classic Libet’s task, participants were alternatively requested to plan when to act (Type I) or to act spontaneously (Type II). Libet observed that trials associated with planning exhibited an even earlier RP onset (about, 1.5 s or more before the onset of the action), while more spontaneous actions were characterized by a late onset (between 500 and 1,000 ms before the onset of the action). Such a simple paradigm clearly does not provide any conclusive evidence regarding the role of distal intentions in daily-life decision-making. However, if a lesson is to be taken, these findings suggest that planning may actually influence the RP, thus supporting both the overall view that the RP is tied to volitional process and the claim that conscious planning (as in type I RP) is associated to specific unconscious neural correlates (Frith and Haggard, 2018).

Second, it has been argued that Libet-type experiments are inadequate to understand motivationally loaded intentions (Lavazza and De Caro, 2010; Mele, 2010). In Libet-type experiments, participants have to repeat identical meaningless movements. Spontaneity in action is limited to deciding when to act, but no specific motivation or reason drives participants’ decision to act at t_1_ or t_2_. By contrast, conceptual models of intentional action are tailored to fit the rich and complex experience of planning, selecting, and executing a wide repertoire of intelligible actions: reasons play an explanatorily role with respect to what a person decides to do and when a person decides to do it.

Such a criticism is well motivated. However, we question the basic idea that non-motivationally loaded intentions would not count as proper intentions. On the one hand, daily actions are often characterized by a lack of clearly appreciable motivations or reasons (e.g., for no specific reason, Mary crosses the street at t_1_ or at t_2_), remaining nonetheless intentional or voluntary. On the other hand, it is unclear whether an increase in the complexity of the choice one has to make (e.g., Peter deciding whether to go to Paris) would translate in measurable changes at the level of the motor pathways enabling intentional actions.

Empirical studies have highlighted a wide range of factors that influence people’s choices. Primarily, anticipated rewards and punishments respectively mediated by the dopaminergic system and fear-learning-related mechanisms, shape our decisions by determining our preferences (Schultz, 1998; Seymour et al., 2007; Robinson et al., 2010). Differences between meaningless and meaningful choices are likely to impact on these circuits rather than on the brain mechanisms that are responsible for motor control, where the RP arises. Consider the following example: Peter has to choose between two possible holiday destinations by selecting between box_1_ (train to Paris) and box_2_ (train to Edinburgh) on the computer screen. Different factors, notably computations of expected costs and benefits associated to the two available options, are expected to determine Peter’s final choice. Depending on Peter’s selection, a motor command will be transmitted to the spinal cord and muscles in order for him to click on the chosen box on the computer screen. Whereas complexity at the level of the decision-making process – where reasons for action and preferences are involved – is likely to impact on the brain mechanisms computing expected rewards and punishments the bodily movement *per se* is comparable to the ones participants execute in Libet-types experiments.

Moreover, some attempts to model whether motivationally loaded intentions impact on the brain mechanisms underlying volitional processes, notably the RP, have been recently made. For example, Khalighinejad and colleagues embedded the Libet’s paradigm in a perceptual decision task in which participants were required to detect the motion of a display of dots toward the left or the right side of the screen (correct answers corresponded to points in the game). In each trial, participants had no clue about when the dots would have started moving to the right or the left side of the screen. If participants did not wish to wait anymore, they had the option to press a skip button and move to the next trial. Voluntary actions were operationalized as self-initiated skip responses to terminate the wait. The reason for acting (i.e., skipping to the next trial) consisted in avoiding potentially long delays before the dots started moving coherently in one direction. Since participants were preliminarily informed that the experiment would have lasted for 1 h, they had a specific reason not to wait for too long before skipping (in order to maximize their final reward). Crucially, Khalighinejad and colleagues were able to observe the RP also for motivated choices, thus potentially extending Libet’s finding to reasons-responsive (Fischer and Ravizza, 1998) actions (Khalighinejad et al., 2018).

#### The Methodological Validity of Libet-Type Experiments

From the methodological perspective, a different interpretation of Libet-type data has recently challenged the view that the RP is a neural marker of the cognitive processes linked to the preparation of bodily movements (Schurger et al., 2012). Schurger and colleagues have argued that the observed changes in the neural activity preceding bodily movements may be ascribed to spontaneous fluctuations in the internal physiological noise. In this case, it would be inappropriate to read the increase in negativity in the electrical activity of the brain as linked to motor preparation. In fact, the timing when the subject executes the action would depend on the timing when the electrical activity reaches a given threshold (i.e., the neural decision to move). A stochastic source of noise in the brain, and not a gradual readable signal corresponding to action planning and preparation, would be responsible for the timing when the action occurs. The model leaves open the possibility that the conscious decision to move *now*, as enabled by some other neural state, still plays a role in action production, coinciding with the time when the sensory-motor threshold is randomly crossed. It remains nonetheless unclear whether this move may counteract epiphenomenalism given that the time when the threshold is crossed is random (what is the causal role of the decision to move now?).

The already mentioned EEG experiment by Khalighinejad and colleagues (Khalighinejad et al., 2018) suggests a possible way to reconcile the classic understanding of the RP as the signal of planning and preparing an action with Schurger’s model of stochastic processes in the brain. Indeed, the experimental findings hint at the specificity of the processes leading to intentional actions, by showing a higher reduction in the signal variability of internally generated actions (compared to externally triggered ones) starting around 1.5 s before the onset of the movement (and consequently before the outcome is produced). To model the data, Khalighinejad and colleagues used a stochastic accumulator model analogous to the one implemented by Schurger et al., 2012. Crucially, the model was able to simulate the RP in the internally generated condition only by introducing, as an additional parameter, a reduction in the amplitude of the noise associated to the preparation of the action. This suggests a possible convergence between Libet’s interpretation of the RP (i.e., the RP as the sign of planning and preparing an action) and the more recent interpretation provided by Schurger and colleagues (i.e., the RP emerges as an artifact due to averaging processes). The RP would therefore reflect stochastic fluctuations of neural activity, but the decrease in variability in the case of internally generated actions would support the presence of a preparatory process prior to actions that are internally generated.

### Internally Generated and Externally Triggered Actions

The above-mentioned criticisms do not undermine the basic claim that intentional actions are preceded by unconscious antecedents. A different way to argue in favor of the role of intentions in action productions consists in acknowledging that intentions may rather play a role that does not coincide with that of initiating the bodily movement. This requires a more integrated model whereby the classic causal theory is reframed in view of the more specific role that intentions may play at different stages of action production. To introduce this view, we start by elucidating the mechanistic model of action production that is central to the cognitive neuroscience of action and volition.

The neuroscience of volition and action broadly distinguishes between voluntary and involuntary behavior, recognizing the former as goal-directed, i.e., aimed at achieving a willed end-state (Fried et al., 2017), and internally generated (Passingham et al., 2010). Engaging in goal-directed behavior implies the capacity to mentally represent a willed end-state (e.g., going home), to compute task-related foreseeable costs (e.g., time and effort in getting home), and to attribute a comparative value to the likely achievement (e.g., getting home vs. spending more time at the office) (Haazebroek and Hommel, 2009). Therefore, such computations take into account a number of variables, including memories of analogous experience and previous errors, understanding of the general norms of behaviors, and estimation of the current state of one’s own body.

The comparison between internally generated (voluntary) and externally triggered (involuntary) actions has proven to be useful to the extent that it provides a set of usable operational definitions for empirical research. Indeed, since Skinner’s work on operant and respondent behavior (Skinner, 1948), this dichotomy has shaped most of the paradigms aimed at casting light on the psychological and brain mechanisms of volition and action. In order to isolate the neuroanatomical structures underlying volitional processes, it is indeed common to compare two experimental conditions. In the externally triggered or instructed one, experimental subjects are requested to react to a specific sensory cue (e.g., “press a button whenever you see a red dot on screen”). By triggering the corresponding bodily movement, the external cue plays the role of the cause of the action. In the internally generated condition, subjects are asked to execute kinematically similar bodily movements, which can be plausibly compared with the ones performed in the externally triggered condition. Crucially, the subject is asked to autonomously set some parameters of the action, e.g., when to make it (e.g., self-paced task), what specific action to perform (e.g., choosing between comparable options), or whether to perform the action or not (e.g., veto experiments) (see Haggard, 2008; Krieghoff et al., 2009). Such a comparison between experimental conditions has revealed differences at the level of the brain areas that are respectively recruited for internally generated and externally driven actions.

Indeed, from the neuroanatomical basis, specific motor circuits in the brain, which are distinct from those sub-serving other forms of behavior, are responsible for enabling voluntary actions. In particular, the decision about what action to perform is taken at the level of the dorsolateral prefrontal cortex (DLPFC), where one out of possible alternatives is selected (Rowe et al., 2000). Once the decision is made, the information about the selected action is forwarded to both the cingulate motor area (CMA), which is in charge of providing the motivational drive that is needed to initiate the bodily movement, and the pre-supplementary motor area (pre-SMA). The execution of all voluntary movements ultimately depends on the primary motor cortex (MI) – receiving afferents from the pre-SMA, the supplementary motor area (SMA), and the premotor cortex (PMC) – which transmits the motor command to the spinal cord and the relevant muscles. Depending on the planned bodily movement, MI would rely on the neural drive provided by the pre-SMA and SMA (for internally initiated actions) or the PMC (for externally driven actions). This dissociation and specialization is supported by empirical evidence, including studies in monkeys that show that selective lesions of the SMA or the PMC can respectively impair internally generated and externally driven motor actions (Okano and Tanji, 1987; Passingham, 1987; Romo and Schultz, 1987; Deiber et al., 1991).

Specific areas in the medial frontal and parietal lobes – according to a gradient from internally guided actions (medial aspect) to externally triggered ones (lateral aspect) – support the features associated to voluntary behavior, such as planning, inhibiting inappropriate courses of action (Filevich et al., 2013), or selecting the option to pursue (Jenkins et al., 2000). Other connected areas are recruited in the coordination and execution of the different phases of action production. Prefrontal regions are involved in action selection and maintenance of the goal throughout the action, while the basal ganglia and the cerebellum participate in the coordination of movement and in cognitively loaded behavior, including planning or reward-based learning (Bostan et al., 2013; Pasquereau and Turner, 2013). By contrast, the PMC, located on the lateral surface of the cortex, immediately anterior to the primary motor cortex, receives inputs from the inferior parietal regions, providing sensory-guidance for the action. Notwithstanding this overall dissociation, both circuits converging in MI contribute to specific aspects of voluntary processes. For example, the circuit involving the PMC, which is generally associated with externally cued actions, might be crucial for stimulus-guided choices between alternatives present in the environment (Shadlen and Newsome, 2001; Haggard, 2008).

To sum up, two distinct motor pathways enable internally initiated and externally triggered (or cued) actions. In the next section, we will delve further into this distinction and its conceptual implications.

### Intentional Actions and Autonomy

By neglecting the role of conscious mental states in defining action, the contrast between internally generated and externally triggered actions does not cast any light on the conceptual distinction between intentional (e.g., Mary is consciously raising her arm in order to ask the speaker a question) and merely voluntary behavior (e.g., Mary is absent-mindedly shifting position while reading a book). At the same time, similarly to conceptual models of action, neuroscientific ones emphasize the role of the causal origin or source of the action. In particular, neurophysiological accounts identify voluntary actions as the class of bodily movements that are internally generated (Passingham et al., 2010). These accounts leave the source of the action underspecified by focusing on what a voluntary action is not. In this sense, they also qualify as *freedom from* models of action. These models readily tap into the intuition that voluntary actions seemingly originate *from within* (i.e., from some internal source of motivation), as opposed to bodily movements that originate *from without* (i.e., from some external clue).

In virtue of its relying on the endogeneity of voluntary actions, the aforementioned distinction dates back to the early stage of the reflection on voluntary behavior in Western culture. In the III book of the *Nicomachean Ethics*, Aristotle similarly distinguishes the concepts of the *voluntary* and the *involuntary* by providing a *freedom from* definition of voluntariness. According to Aristotle’s definition, some features must apply for the action to be deemed as involuntary (Aristotle, 2000). One consists in the external origin of the bodily movement: the action is involuntarily when the movement originates *from without*, while the agent remains *passive* with respect to it. Interestingly, in this context Aristotle hints at a gradualist understanding of agency, by arguing that human actions can be of a mixed or composite nature. This is illustrated by the case of a cargo that is willingly thrown overboard to save the seamen’s life during a storm. In this case, the origin of the action is external (i.e., the necessity to lighten the boat) and no clearly foreseeable alternative is at hand. Nonetheless, the bodily movement (i.e., the guided throwing of the cargo) is still willingly executed. Borrowing a felicitous expression by Gold and Shadlen (2007), voluntary actions are marked by a *freedom from immediacy* (i.e., from the immediate reaction to an externally imposed drive) that involuntary or mixed actions entirely or partially lack.

As emphasized by Aristotelian mixed actions, the stipulation of a sharp dichotomy between voluntary and involuntary actions may overshadow the fact that most of our voluntary goal-directed behaviors are (also) stimulus driven, to the extent that they engage with, and respond to, specific features of the environment. It would be therefore too simplistic to assume that the pair internally generated/externally driven actions maps into the pair voluntary/involuntary actions. Consider a situation where Mary starts driving her car with the purpose of getting home from work and stops in front of the traffic light. In pushing on the gas when she sees the green light, Mary is responding to an external cue. At the same time, she is acting voluntarily in a coordinated effort whose nature results from the combination of the distal goal (i.e., getting home from work), the instrumental bodily movements that are needed in view of the goal (e.g., pushing on the gas), and the contingent constraints that are in place (e.g., the rules of the road, the specific route she took, the mechanics of the car). A wide array of cues determines the goal that Mary selects and the bodily movements that the achievement of the goal requires.

We suggest that the distinction between voluntary/involuntary actions does not ultimately depend on whether the movement is initiated by an external cue or not. More deeply, this comparison hints at the degree with which the agent *autonomously* contributes to solving a task or achieving a goal. The notion of *autonomy* refers to the information the agent has to generate and the decisions she has to make in order to carry out the action. Whereas involuntary movements are automatic responses to preexisting sources of information, when it comes to voluntary behavior preexistent inputs are not sufficient to determine the course of the action.

As Aristotelian mixed actions show, the agent’s behaviors are located along the *continuum* that goes from reflexes to full-fledged voluntary behavior (Haggard, 2014). To illustrate this point, consider the following two variants to Mary’s case. In case_1_, Mary relies on the green light in order to decide when to initiate the action; in case_2_, the traffic light happens to be broken so that it depends on Mary when to initiate the action, integrating different sources of information. In these two cases, the willed-end state is analogous (e.g., getting home from work), but the contribution of the agent is different to the extent that, in case_2_, she cannot entirely rely on externally available inputs about when to push on the gas and has to autonomously produce the information that is needed to fulfill the task.

### Intentional Actions and Causality

In the section “Intentional Actions and Autonomy,” we argued that the comparison between internally generated and externally triggered actions does not fully capture the distinction between intentional, voluntary and involuntary behaviors. We partially integrated this distinction by suggesting that endogeneity is to be understood in terms of the subject being partially autonomous in generating the information needed to solve a task or achieve a goal. In this section, we move a step forward in clarifying how to understand autonomy in relation to action production. Indeed, it would be misleading to conclude that, in the absence of an identifiable cue, the action is underdetermined or happens without a cause. A causal theory of action can appropriately account for intentional behavior to the extent that it can explain it as the resultant of multiple integrated causal vectors.

The subjective feeling of acting without a cause is likely to play a major role in our pre-theoretical understanding of intentional action. By contrast, the agent’s behavior is routinely constrained by memories of previous similar experiences and other occurring mental states, which limit the range of available options working as causes of the agent’s behavior. Going back to our example, Mary’s autonomous decision about when to push on the gas is concurrently determined by her overall goal (e.g., getting home as soon as possible) and the inputs deriving from her experience as a driver (e.g., waiting until the pedestrians cross the street, avoid crossing when the traffic light is red).

However, intentional actions seemingly imply a specific moment of deliberation or choice in order to initiate a movement. The concept of *having a choice* is central to the metaphysical discussion about free will and determinism (van Inwagen, 1983; Pereboom, 2014), usually in terms of whether individuals can actually do otherwise, and thus have a choice, when they decide what to do. In this article, we set aside the question of whether people have a choice in the metaphysical sense. Indeed, we adopt a more limited concept of *choice*: when it comes to planned behavior (e.g., Peter is deciding at t_0_ whether to go to Paris at t_1_), individuals are able to engage in goal-directed reasoning, express preferences about seemingly available options, and finally act upon those preferences. Alfred Mele’s notion of psychological autonomy is in line with these *desiderata*. According to Mele, compatibilist psychological autonomy is fulfilled when the following three conditions jointly hold: (1) the agent lacks compelling or coercive motivational states; (2) the agent’s beliefs are conducive to informed deliberation; (3) the agent is a reliable deliberator (Mele, 1995).

Options in the environment are associated to a specific valence, which is explicitly or implicitly taken into account by the agent in making her choices. This ongoing, often automatic, integration of different inputs in setting up plans and acting upon them contributes to blurring the distinction between internally generated and externally triggered actions. For example, previously rewarded goals become more likely to be chosen again in the future, because of progressive reinforcement (Cushman and Morris, 2015). For instance, had Peter’s holiday been successful, he might be more prone to choose Paris as a destination in the future. This implies the additional capacity to monitor successful links between actions and outcomes and to store them in memory for future use. This view easily accommodates the possibility that, after making the decision to go to Paris, Peter inferentially assumes that his conscious desire to go to Paris causes his decision, ignoring the other (unconscious) determinants of his choices. Limitations in the agent’s introspective power and metacognitive ability do not undermine her being aware of ongoing deliberative processes that are expected to have a causal impact on the subsequent behavior.

In acting upon different internal and external causal vectors, humans display a high level of sophistication and flexibility at the level of the means the agent can deploy and at the level of the ends she can achieve. If Mary’s goal is to interact with the speaker at the end of the talk, she will be more or less successful depending on her ability to adaptively modulate her behavior based on external constraints, e.g., in terms of choosing the appropriate timing, of whether to ask her question or not, and of what specific question to ask. If different opportunities arise, Mary may even set out a different goal and act accordingly. These choices rely on mental simulation of the space of action and of possible counterfactual scenarios. In navigating the environment, agents are constantly exposed to a number of cues that modulate their behavioral responses. Within a causalist model of action production, intentional and voluntary actions are therefore to be understood as the resultant of a number of integrated causal vectors.

Hence, two types of abilities are crucial for the agent to achieve a goal. On the one hand, at the level of means, the agent needs the instrumental knowledge that is required in order to translate an abstract goal into an effective course of action, including the capacity to select a bodily movement that is appropriate to the task (Ghahramani and Wolpert, 1997; Rowe and Passingham, 2001). On the other hand, at the level of ends, the agent must set her own goals based on the information provided by the combination of existing internal and external cues. Whereas intentional action presumably requires awareness or conscious monitoring, other forms of voluntary behavior are cognitively less demanding. Research on habitual actions has suggested that, when possible in virtue of the task, subjects can switch into a sort of automatic mode that does not imply cognitive control and is insensitive to outcome devaluation (Dickinson and Weiskrantz, 1985; Graybiel, 2008; Dezfouli et al., 2014). In line with our gradualist view, habitual actions can be goal-directed or purposive (e.g., Mary is shifting position to be more comfortable), despite lacking the conscious awareness or online monitoring that characterize intentional behavior.

Recent experimental evidence in the field of decision-making has suggested that the brain flexibly exploits both goal-directed and habitual systems within the same task. Goal-directed decision-making relies on the individual ability to build models of the environment and of its dynamic evolution, in order to simulate future consequences of a decision. This simulation process is computationally expensive, prone to error and time consuming. Crucially, subjects have been shown to switch from one system to the other depending on ongoing task demands. When possible, subjects can thus revert to habitual decision-making, which has the advantage of being computationally simpler and does not require planning. The choice of the goal directed vs. habitual mode depends on a number of variables that influence decision-making, such as time, working memory load, opportunity cost, or stress (Keramati et al., 2016).

### Intentional Actions and Control

In the section Intentional Actions and Causality, we suggested a revision of the causal theory that acknowledges that autonomous actions result from the flexible integration of different causal vectors. This section sets out to introduce one further feature of voluntary behavior, namely action control. Within the framework of the causal theory, control is understood in terms of the agent’s capacity to achieve a given end-state through her bodily movements, by acting as she wanted to act.

However, whereas action control is crucial for voluntary behavior, the nature of the control-conveying property eludes explanation. In particular, it is unclear whether action control requires conscious awareness. A lively debate in the literature on skilled actions contends that there are situations in which automatic behaviors, lacking conscious monitoring or awareness, allow for more accurate and quicker (and thus more controlled) responses in rapidly changing environments (Shepherd, 2017). At the same time, it remains questionable whether low-level, automatic processes can account for the fine-grained movements featuring in skilled actions (Fridland, 2016). In cognate research fields, it has also been proposed that control can be achieved in the absence of conscious awareness. For example, according to Dienes and Perner’s *cold control theory*, the agent’s successful response (i.e., where the subject correctly responds to the provided instructions) to hypnotic suggestions depends on forming an intention without the agent being aware of having formed that intention (Dienes and Perner, 2007).

It seems that we need a distinction between two types of action control. The first consists in the agent’s ability to effectively do what she wants to do, e.g., the ability to stick to one’s own plans. In this sense, action control clearly requires a certain degree of conscious awareness (i.e., it would not make sense to say that the agent was able to do what she planned to do without the agent being at least partially aware of what she planned to do). The second consists in the agent’s ability to engage in guided behaviors. In this latter case, it is not necessary that the agent is aware of what she wanted to do. However, in both cases control implies a certain degree of matching between the conscious (or, possibly, unconscious) intention and the corresponding behavior. We suggest that a gradualist approach might be more advantageous in both cases.

For what concerns the first type, Joshua Shepherd has recently argued that action control is achieved when the agent acts in accordance with her intentions, in spite of extenuating circumstances that pull her in other directions (Shepherd, 2014). Control would thus indicate the agent’s resilience to external obstacles, so that the agent can be more or less in control of her action depending on the extent to which she would be able to do what she wanted to do, in presence of potential obstacles. In the case of deviant causal chain, the agent chancily achieved the outcome she wanted to achieve, thus lacking control across extenuating circumstances. In daily life, resilience, as tied to action control, seemingly implies that the agent’s is able to successfully adapt means and ends to variations in the external environment. When it comes to the second type, control implies action guidance, independently of whether the action depends on what the agent wanted to do. Even in this case, the action can be more or less guided depending on the extent to which the behavioral response matches the (unconscious) intention or the externally driven (e.g., as in the case of hypnosis) instructions.

This variety of types of voluntary and intentional behavior requires a much more complex conceptual framework than the one originally provided by the causal theory. We suggested that the underlying notions of intentional agency and control still require a causal approach to the extent that this is able to integrate the contribution of multiple consciously and unconsciously processed causal vectors. Theories of the cognitive architecture of the prefrontal cortex seem to adequately match this multilayered view. In particular, it has been proposed that control or guidance is practically achieved by means of the integration of the abstract level of goals and the instrumental level of fine-grained bodily movements. Such an integration might be sub-served by hierarchical processes linking abstract goals with executive motor commands. In particular, the cascade model proposed by Koechlin and Colleagues (Koechlin et al., 2003; Koechlin and Summerfield, 2007. For a more horizontal model of action control, see Uithol et al., 2014; Schurger and Uithol, 2015) describes action control as a hierarchically ordered process made possible by a cascade of top-down control from rostral to caudal lateral prefrontal cortex (LPFC) and premotor regions, with anterior areas devoted to deliberative, abstract, temporally extended action control. Different areas of the control network in the LPFC would be responsible for executive control, globally understood as the capacity to select specific actions in view of a goal, thus resolving the entropy or competition between different action representations. Throughout this multilayered system, executive control and action coordination would be nevertheless guaranteed by the tight integration of information across the multiple specialized prefrontal regions, varying according to different degrees of flexibility and capacity of abstraction.

## Conclusion

There are few concepts as relevant as that of intentional action in shaping our sense of self and the interaction with the environment. At the same time, few concepts are so elusive. Classic models in the philosophy and cognitive science of action struggle to offer a positive description (*freedom to*) of intentional agency. The goal of this article was to bridge the gap between disciplinary approaches in order to frame a conceptually rich and empirically sound model for agency. We settled for a graded notion of intentional agency as a form of goal-directed behavior whereby the agent has the ability to introspect upon the conscious mental states that are seen as the causes of one’s own actions. Furthermore, we relocated intentional action within the wider context of voluntary behavior, insisting on some key features, notably autonomy, flexibility in the integration of distinct causal vectors, and control.

Several questions remain open. Perhaps the most fascinating ones concern the match between what we named as the *subjective* and the *objective* sides of agency. In our view, the combination of conceptual analysis and empirical investigation is likely to continue playing a key role for this research field.

## Author Contributions

SB drafted the manuscript. PH provided critical revisions. All authors approved the final version of the manuscript for submission.

### Conflict of Interest Statement

The authors declare that the research was conducted in the absence of any commercial or financial relationships that could be construed as a potential conflict of interest.
